# Comparison of elicitor-based effects on metabolic responses of *Taxus* × *media* hairy roots in perfluorodecalin-supported two-phase culture system

**DOI:** 10.1007/s00299-018-2351-0

**Published:** 2018-11-07

**Authors:** K. Sykłowska-Baranek, W. Rymaszewski, M. Gaweł, P. Rokicki, M. Pilarek, M. Grech-Baran, J. Hennig, A. Pietrosiuk

**Affiliations:** 1 Department of Pharmaceutical Biology and Medicinal Plant Biotechnology, Faculty of Pharmacy with the Laboratory Medicine Division, Medical University of Warsaw, 1 Banacha Str, 02-097 Warsaw, Poland; 2 Institute of Biochemistry and Biophysics, Laboratory of Plant Pathogenesis, Polish Academy of Sciences, 5A Pawińskiego Str, 02-106 Warsaw, Poland; 3 Faculty of Chemical and Process Engineering, Warsaw University of Technology, Waryńskiego 1, 00-645 Warsaw, Poland

**Keywords:** *Taxus*, Hairy roots, Elicitation, Perfluorodecalin, Gene expression

## Abstract

**Key message:**

Two lines of *Taxus* × *media* hairy roots harbouring or not the *TXS* transgene demonstrated diverse gene expression and taxane yield during cultivation in PFD-supported two liquid-phase culture system.

**Abstract:**

Two lines of *Taxus* × *media* hairy roots were subjected to single or twice-repeated supplementation with methyl jasmonate, sodium nitroprusside, l-phenylalanine, and sucrose feeding. One line harboured transgene of taxadiene synthase (ATMA), while the second (KT) did not. Both hairy root lines were cultured in two-phase culture systems containing perfluorodecalin (PFD) in aerated or degassed form. The relationship between *TXS* (taxadiene synthase), *BAPT* (baccatin III: 3-amino, 3-phenylpropanoyltransferase), and *DBTNBT* (3′-*N*-debenzoyl-2-deoxytaxol-*N*-benzoyltransferase) genes and taxane production was analysed. The ATMA and KT lines differed in their potential for taxane accumulation, secretion, and taxane profile. In ATMA biomass, both paclitaxel and baccatin III were detected, while in KT roots only paclitaxel. The most suitable conditions for taxane production for ATMA roots were found in single-elicited supported with PFD-degassed cultures (2 473.29 ± 263.85 µg/g DW), whereas in KT roots in single-elicited cultures with PFD-aerated (470.08 ± 25.15 µg/g DW). The extracellular levels of paclitaxel never exceeded 10% for ATMA roots, while for KT increased up to 76%. The gene expression profile was determined in single-elicited cultures supported with PFD-degassed, where in ATMA roots, the highest taxane yield was obtained, while in KT the lowest one. The gene expression pattern in both investigated root lines differed substantially what resulted in taxane yield characterized particular lines. The highest co-expression of *TXS, BAPT* and *DBTNBT* genes noted for ATMA roots harvested 48 h after elicitation corresponded with their higher ability for taxane production in comparison with the effects observed for KT roots.

**Electronic supplementary material:**

The online version of this article (10.1007/s00299-018-2351-0) contains supplementary material, which is available to authorized users.

## Introduction

Taxane-type compounds are well-known drug agents used in anti-cancer therapies against tumors of ovaries, breast, prostate and lung cancers, Kaposi’s sarcoma, squamous cell carcinoma of head and neck, and many others (Yared and Tkaczuk 2012). Based on constantly rising market-demand for paclitaxel and other taxanes, various biotechnological platforms for their production have been developed (which was extensively discussed in Tabata 2004; Vongpaseuth and Roberts 2007; Frense 2007; Maheshwari et al. 2008; Expósito et al. 2009; Sabater-Jara et al. 2010; Malik et al. 2011; Onrubia et al. 2013a; Cusido et al. 2014). The authors summarized a variety of strategies leading to enhance taxane in vitro production in biomass of various *Taxus* species. The most effective seemed to be the elicitation with methyl jasmonate (MJ). An immense effort was undertaken to elucidate MJ molecular mechanism of action in *Taxus* cell suspension cultures, which led to revealing up-regulation of the majority of genes engaged in metabolic pathways leading to taxane biosynthesis (Nims et al. 2006; Exposito et al. 2010; Onrubia et al. 2010, 2013b; Patil et al. 2012; Sabater-Jara et al. 2014). These investigations unraveled the 1–7-day delay between a time course for mRNA accumulation of the known taxane biosynthetic genes and taxane production (Nims et al. 2006; Lenka et al. 2012). Moreover, the profiling of transcripts after MJ elicitation of cells in suspension cultures of various *Taxus* species, demonstrated up-regulation not only of genes involved in taxane biosynthesis, but also genes engaged in the stimulation of plant hormone and phenylpropanoid biosynthesis, MJ-signaling, taxane transport, degradation, as well as transcriptional regulation (Lenka et al. 2012; Li et al. 2012; Sun et al. 2013). Despite the enormous efforts undertaken for a better understanding of taxane metabolism in plant biomass from in vitro cultures, the taxane biosynthesis rate-limiting steps remained still unidentified, although the late pathway steps seems to be potentially and significantly rate-influencing steps in paclitaxel production (Nims et al. 2006; Vongpaseuth and Roberts 2007; Sabater-Jara et al. 2014; Cusido et al. 2014).

Other factors recognized as suppressing taxane yield are feedback inhibition and taxane degradation. To overcome these phenomena, as well as facilitating product recovery, the two-phase systems containing organic layer non-mixed with aqueous-based culture medium, for in situ extraction were developed. The major disadvantage of this approach is the negative influence on biomass growth rate (Sabater-Jara et al. 2010; Wilson and Roberts 2012; Cai et al. 2012). In our approach, we propose the application of biologically inert perfluorodecalin, as a second liquid phase of respiratory gas carrier, and in situ extractant as well (Pilarek 2014; Sykłowska-Baranek et al. 2014, 2015b). Liquid perfluorochemicals (PFCs) dissolve gases according to the Henry’s Law, and the gas transfer rate into PFCs increases linearly with the partial pressure of a component in the gaseous phase (Castro and Briceno 2010; Sobieszuk and Pilarek 2012). Importantly, PFCs are immiscible with aqueous media, so they create a separate phase, below the aqueous phase, on the bottom of the culture flask/vessel. Both these facts cause that in PFC-supported culture system, the additional interfacial area (PFC/medium) for mass transfer appears independently of typical medium/air-phase interface, what is recognized as the possibility for in situ mass transfer intensification in the culture system.

Up to now, the boosting effects of range of elicitors on some taxane biosynthesis genes in relation with taxane production were investigated. The following elicitors were examined in *Taxus* spp. cell suspension cultures: MJ (Nims et al. 2006; Exposito et al. 2010), MJ individually or in combination with vanadyl sulphate (Onrubia et al. 2010), MJ or coronatine (Onrubia et al. 2013b), and MJ jointed with cyclodextrins (Sabater-Jara et al. 2014). The results of the investigations mentioned above indicated that the possible rate-limiting steps in taxane biosynthesis are controlled by genes of the late pathway: *BAPT*—baccatin III: 3-amino,3-phenylpropanoyltransferase the enzyme which integrates baccatin III with phenylisoserine to 3′-*N*-debenzoyl-2-deoxytaxol (Walker et al. 2002a) and *DBTNBT—*3′-*N*-debenzoyl-2-deoxytaxol-*N*-benzoyltransferase, the enzyme produces 2′-deoxytaxol through ligation of benzoyl CoA and 3′-*N*-debenzoyl-2-deoxytaxol (Walker et al. 2002b).

Regardless of very few data on taxane metabolic pathways in suspended cells, there are still any report published which were focused on detailed investigation of taxane biosynthesis genes in *Taxus* spp. hairy root in vitro systems. However, previously, it has been reported that among various approaches used to enhance taxane production in *Taxus* × *media* hairy roots, the most promising seems to be the simultaneous application of MJ and two-phase culture system containing PFD as in situ extractant and liquid gas carrier (Sykłowska-Baranek et al. 2015b). Next, the increase of taxane yield was achieved by the joint action of MJ and sodium nitroprusside (SNP, as NO donor) combined with l-phenylanine (PHEN) as biosynthesis precursor and sucrose feeding (Sykłowska-Baranek et al. 2015a).

The novelty of the current study is the comparison of the effect of single or twice-repeated elicitation with MJ, sodium nitroprusside, l-phenylalanine, and additional sucrose, in the two-phase culture systems in situ integrating aqueous phase of culture medium and liquid phase of PFC (perfluorinated solvent, as well as liquid gas carrier), on the yield of taxane and elucidation of molecular mode of action of applied elicitor agents. To our knowledge, this is the first report on the gene expression profiling in *Taxus* spp. hairy root cultures in response to elicitor treatment in situ performed in two-phase culture system.

## Materials and methods

### Hairy root cultures

Two lines of *Taxus* × *media* hairy roots were investigated. The first root line, KT, was obtained by 8-week-old seedlings transformation performed with *Agrobacterium tumefaciens* strain LBA 9402 (Furmanowa and Sykłowska-Baranek 2000). The second root line, ATMA, carries *TXS* transgene from *T. baccata* (GenBank accession: AY424738), and was originally developed as a result of transformation of 10-year-old *Taxus* plantlets cultivated in vitro with the *A. tumefaciens* C58C1 strain (Sykłowska-Baranek et al. 2015b). The hairy roots were maintained in a hormone-free liquid DCR medium modified by increased concentration of MgSO_4_ (400 mg/l) (DCR-M) (Syklowska-Baranek et al. 2009). Both types of hairy roots were routinely subcultured every 4 weeks. The cultures were carried out at 23 ± 1 °C in the dark, on the INFORS AG TR 250 shaker (Switzerland) operating at 105 rpm. All hairy root cultures were carried out in 250 ml Erlenmeyer flasks containing 35 ml of DCR-M medium for 49 days.

### Elicitation experiments

The eight variants of the elicited cultures were carried out and the results were compared to the control untreated cultures referentially maintained in DCR-M medium without any supplementation. The mixture of compounds used to elicit taxane production was composed of: elicitors MJ (100 µM, Sigma-Aldrich, Poland) and SNP (10 µM, Sigma-Aldrich, Poland), precursor PHEN (100 µM, Sigma-Aldrich, Poland), and sucrose (30 g/l, Avantor Performance Materials Poland S.A.). Such mixture is called “elicitors” in further text. The elicitors were added to the media under sterile conditions in a laminar cabinet. The sequence of PFD and elicitors application has been presented in Table 1S. In all experiments, inoculum in a form of 0.5 ± 0.05 g of 28-day-old roots transferred to the fresh DCR-M medium has been used. On the 14th day of the experiment, some culture variants has been supplemented with PFD-degassed or PFD-aerated. The elicitation has been performed at one time point for all culture systems—on the 28th day of culture, or two times, on the 28th day, as well as 35th day. The following 8 culture variants were proceeded: (1) single-elicited cultures without PFD; (2) twice-elicited cultures without PFD; (3) unelicited cultures with PFD-aerated; (4) single-elicited cultures with PFD-aerated; (5) twice-elicited cultures with PFD-aerated; (6) unelicited cultures with PFD-degassed; (7) single-elicited cultures with PFD-degassed; and (8) twice-elicited cultures with PFD-degassed. PFD (C_10_F_18_; 98% equimolar mixture of *cis*-/*trans*-isomers; ABCR GmbH & Co. KG, Karlsruhe, Germany) was prepared and added to the cultures, as described before (Sykłowska-Baranek et al. 2014), autoclaved at 121 °C for 20 min., and if necessary (in the case of PFD-aerated), it was aseptically saturated with atmospheric air for 15 min.

**Table 1:** Primer sequences used to amplify the genes by qRT-PCR

Gene	Primer sequence	Amplicon size	GenBank accession
*18S*	Forward: 5′-GTGACGGGTGACGGAGAATTAG-3′	144	AY544989.1
	Reverse: 5′-CGTGAGCCCAGTATTGTTATTTATTGTC-3′		
*β-Tubulin*	Forward: 5′-GGCTTTCTTGCACTGGTACAC-3′	164	AB918700.1
	Forward: 5′-CATCTCCTGAAACTACCGACTC-3′		
*TXS*	Forward: 5′-TGCGTGCCCTGTATGTATTCC-3′	79	AY461450.2
	Reverse: 5′-GACCGATTCCGAGATGCTCAAT-3′		
*BAPT*	Forward 5′-CAGGTTCGTTAGCAGAGTTCCA-3′	106	AY563630.1
	Reverse 5′-ATGTTGTCAATGGCGGAGAGA-3′		
*DBTNBT*	Forward 5′-CGATGCGTCCACCTCCAATAG-3′	141	AY563629.1
	Reverse 5′-CCAATGCTGCTACGACCTCAA-3′		

The samples were harvested on the 14th, 28th, 35th, 42nd, and 49th day of culture from the control cultures, and from the elicited and/or PFD-supported culture variants at adequate time points. Biomass of hairy roots were then separated from the liquid phase/s, gently pressed on filter paper, and weighed to obtain fresh weight (FW). After lyophilization (lyophilizer Christ ALPHA1-4 LSC, Germany), the dry weight was recorded (DW).

### Taxane determination

The concentration of 10-deacetylbaccatin III, baccatin III, 10-deacetyltaxol, 7-epi-10-taxol paclitaxel, paclitaxel and taxol C was investigated. All standard compounds were produced by ChromaDex (USA), and purchased in LCG Standards (Poland). The samples were prepared from lyophilized root biomass, aqueous phase of media, as well as from PFD. The extraction of taxanes from root biomass, culture medium and PFD, as well as the process of sample cleaning, and HPLC-UV-DAD analysis; all of them were performed as described earlier (Sykłowska-Baranek et al. 2015b), according to the methods developed by Theodoridis et al. (1998a, b), respectively. The taxanes were identified and quantified at 227 nm. The peaks were assigned by spiking the samples with individual chemical standard and comparing the retention times and UV spectra as well. All chemicals for HPLC analysis were purchased in Sigma-Aldrich (Poland). All experiments were carried out in triplicate and the statistical significance between means was assessed using the Kruskal–Wallis one-way analysis of variance preformed with the STATISTICA 13.1 PL software. A probability of *p* < 0.05 has been considered as significant.

### Quantitative real-time PCR

For analysis of gene expression in hairy roots of ATMA and KT lines, the biomass was harvested from cultures supplemented with PFD-degassed and subjected to single elicitation. The samples were harvested before elicitation—time point 0 (28th day of culture, control culture), and after 6 h, 12 h, 24 h, 48 h, and 1 and 2 weeks after elicitors addition into the culture system. The influence of elicitors on the expression levels of three genes was examined and investigated: *TXS, BAPT*, and *DBTNBT*. The total RNA was isolated form frozen roots (0.1 g FW) using the modified method described by Chomczynski and Sacchi (1987). Before homogenization 2% PVPP (Sigma-Aldrich, Poland), 2% β-mercaptoethanol (Sigma-Aldrich, Poland), and 1% SDS (Sigma-Aldrich, Poland) were added to the sample. One 1 µg of the total RNA from each sample was subjected to reverse transcription using the cDNA RevertAid First Strand cDNA Synthesis Kit and random hexamer primer (both from Thermofisher Scientific, USA) according to the manufacturer’s instruction. Quantitative real-time polymerase chain reaction (qRT-PCR) was performed using SYBR Green PCR Mastermix (Roche, Germany) in a 384-well platform system (LightCycler 480 Instrument, Roche, Germany). Sequence-specific primers were designed with Primer3 software version 0.4.0 (Table 1). Data were analysed with the LightCycler 480 Software release 1.5.0 SP3 (Roche, Germany). The qRT-PCR was performed using a 20-fold cDNA dilution as a template. The parameters of qRT-PCR was as follows: initial denaturation at 94 °C for 10 min, followed by 40 cycles at 94 °C for 30 s, 58 °C for 25 s, 72 °C for 25 s, and then a final extension at 72 °C for 7 min. The transcript level of each gene was normalized against reference genes: 18S rDNA and β-tubulin using the ΔΔCt method with PCR efficiency correction. The reported values are an average of four biological replicates and three technical replicates.

## Results and discussion

### The influence of culture conditions on hairy root biomass accumulation

**Fig. 1:**
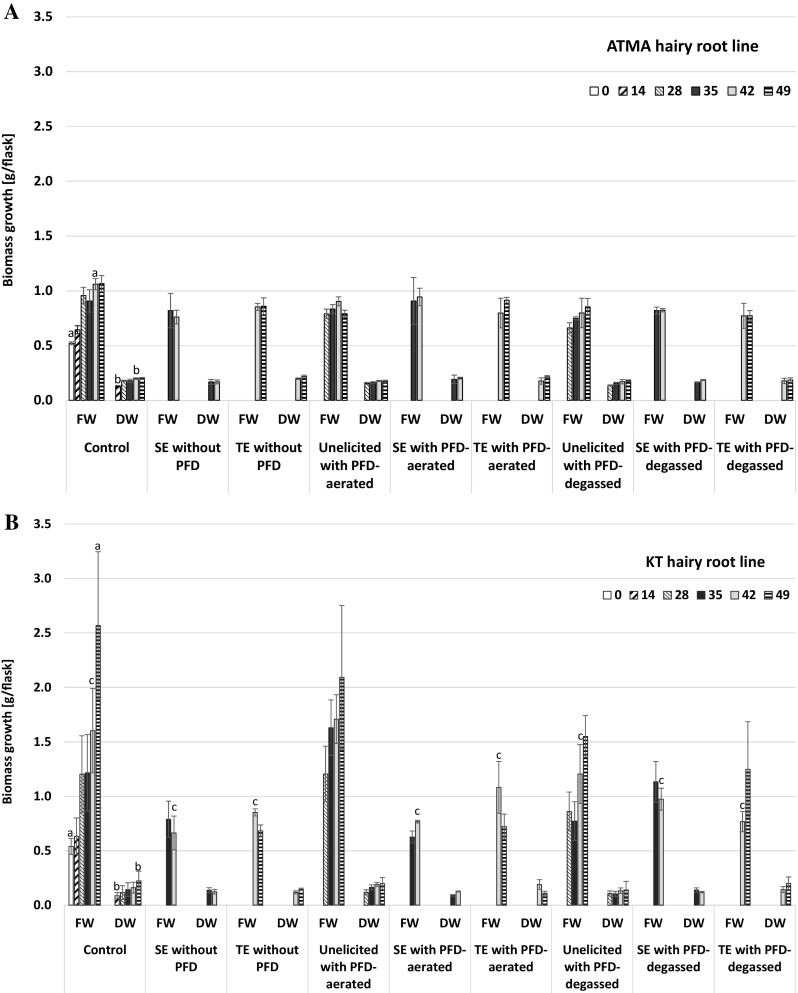
Biomass growth (g/flask) in ATMA (harbouring *TXS* transgene) and KT (without *TXS* transgene) hairy root lines of *Taxus* × *media* cultivated in various culture variants: control—untreated cultures; single–elicited (SE) cultures without PFD; twice-elicited (TE) cultures without PFD; unelicited cultures with PFD-aerated; single-elicited cultures with PFD-aerated; twice-elicited cultures with PFD-aerated; unelicited cultures with PFD-degassed; single-elicited cultures with PFD-degassed; twice-elicited cultures with PFD-degassed. Data represents mean of three replicates ± SD; the same letters indicate means statistically different (*p* < 0.05)

The growth of two hairy root lines ATMA and KT was examined in elicited and unelicited cultures, both supported or not with PFD degassed or PFD aerated. The results were referenced to untreated control cultures. In the case of both investigated root lines, the highest accumulation of fresh biomass was noted in control cultures, at the end of culture (Fig. 1a, b). However, their growth pattern was different. In the case of ATMA line, biomass started to grow from the inoculation day until the 28th day, and from this time point, the stationary growth phase was observed. In the case of KT line, the stationary phase has not been reached. The increase in dry biomass observed in cultures of both hairy root lines followed the fresh biomass accumulation pattern. In the control cultures, the values of final fresh biomass, as well final dry biomass achieved by KT roots were higher than for ATMA roots, and these differences were statistically significant (*p* > 0.05). The distinct discrepancy in the fresh biomass obtained at the end of culture could be attributed to the post-harvesting manipulation and insufficient medium removal, what leads to irreproducible water content in samples. Hence, the dry biomass increase seems to be more suitable parameter for quantitative comparison of the growth capacity of the both investigated hairy root lines. In the ATMA root line, the highest growth reduction, by 15%, was noted in single-elicited cultures without PFD, followed by 14% and 11% decrease in dry biomass, respectively, in unelicited cultures supported with PFD-aerated or PFD-degassed (Fig. 1). However, growth stimulation was also demonstrated, in PFD-aerated supplemented culture variants with single or twice-repeated elicitation by 2% and 7%, respectively. In cultures of KT roots, the most pronounced growth inhibition, by a ratio of 52%, was noted in a single-elicited culture supported with PFD-aerated. The dry biomass accumulation of KT roots was also noticeably reduced in systems with PFD-degassed, without elicitation and in single-elicited cultures without PFD, by 37% and 35%, respectively. The results described in the current study stay in compliance with the results reported earlier by Sykłowska-Baranek et al. (2015a), where the similar elicitors mixture caused the reduction of dry biomass accumulation of ATMA hairy roots by 17%. The effect of PFD supplementation under the conditions of the current examinations depends on hairy root line and the type of PFD applied into the culture system, that is PDF-degassed or PFD-aerated. In ATMA root line cultures, the biomass accumulation was not affected by the presence of the PFD phase, irrespectively in degassed or aerated form (Fig. 1a). Whereas in cultures of KT roots, in elicited variants supported with PFD-aerated or PFD-degassed, the root growth was slightly higher than in elicited variants without any PFD (Fig. 1b). In previously investigated ATMA root cultures performed in two-phase systems supported with PFD-aerated or degassed and elicited with MJ, a slight root growth reduction by 10% and 1%, respectively, has been observed. At the same time, in solely MJ-elicited cultures, hairy root growth was inhibited by 13% (Sykłowska-Baranek et al. 2015b). The majority of organic phases applied into in vitro culture systems for bioprocesses with *Taxus* spp. biomass exhibited a significantly detrimental effects on biomass growth (Sabater-Jara et al. 2010; Wilson and Roberts 2012; Cai et al. 2012; Malik et al. 2013). However, the results of the current study indicate on advantages of the two-phase system with biologically inert PFD (Pilarek 2014) applied in situ as biocompatible solvent.

On the molecular level, it was demonstrated that in various *Taxus* spp. cell suspension cultures, the exogenous application of MJ resulted in the down-regulation of genes engaged in primary metabolism. At the same time the up-regulation of genes expression involved not only in taxane biosynthesis, but also in other metabolic pathways including diterpene and triterpene backbone biosynthesis, genes involved in the regulation of gene expression (Sun et al. 2013), jasmonate signaling transduction pathways, biosynthesis of hormones (Li et al. 2012), taxane transport, or degradation (Lenka et al. 2012) was observed.

Exposito et al. (2010) investigating two transformed lines of *T*. × *media* cell suspension cultures, RolC carrying only *ROL* genes of *A. rhizogenes* and TXS carrying *ROL* genes and *TXS* transgene, showed 26% and 28% growth decrease, respectively, after MJ addition. Such effect is different from results observed in the current study, because the growth of KT roots (carrying *ROL* genes without *TXS* transgene) was more susceptible to applied culture conditions than ATMA roots (carrying *ROL* genes and *TXS* transgene). Next, Onrubia et al. (2013b) noted stronger biomass suppression in response to MJ application in comparison with unelicited and coronatine-treated cultures in the case of cultures of the same TXS cell lines. In the majority of research dealing with the response of *Taxus* spp. cells, tissues and organ cultures to MJ treatment, the growth retardation was reported (Wilson and Roberts 2012; Sabater-Jara et al. 2010) what is attributed to the accumulation of paclitaxel and/or other taxanes in the cultures both intra- and extracellulary (Kim et al. 2004; Expósito et al. 2009). The detrimental effect of MJ exhibited in *Taxus* cell growth could be explained by the impaired G1/S transition, an increase in G0/G1 phase cells, as well as by decrease in the number of dividing cells (Patil et al. 2014). Moreover, Sun et al. (2013) observed the decrease in gene expression engaged in cell cycle progression, DNA replication, meiosis and mismatch repair in MJ-elicited cell suspension cultures of *T*. × *media*. However, in the case of conditions of the current experiments, the detrimental effect of MJ application in culture variants supplemented with PFD was diminished in comparison with single-phase culture variants which corroborates with results described by Sabater-Jara et al. (2014), where authors noted lower reduction of cell growth exposed to MJ and interpreted this as a result of prior application of cyclodextrins, to the culture media.

### Taxane production

Among six investigated taxanes only paclitaxel and baccatin III were found in ATMA roots, while in KT roots only paclitaxel has been detected. Both compared hairy root lines differed substantially in their capacity for taxane production. The elicitation step was essential in inducing taxane biosynthesis in ATMA roots, whereas for KT roots, paclitaxel presence was determined also in unelicited culture variants (Tables 2, 3, 4). In ATMA roots’ cultures, irrespectively PFD treatment regime, paclitaxel was revealed not only in the root biomass but also in the aqueous phase of culture medium and in PFD as well (Table 2). In the cultures of KT roots, paclitaxel was detected mostly in root biomass, but it was also sporadically found in the medium, solely in twice-elicited variants, 1 week after repeated elicitation (on the 42nd day) (Table 3). Baccatin III produced in ATMA roots was detected uniquely in root biomass and in culture variants containing PFD phases (Table 2).

**Table 2:** Paclitaxel content [µg/flask] determined in *Taxus* × *media* ATMA hairy root line cultures subjected to elicitation

Culture variant	Single-elicited cultures without PFD						Twice-elicited cultures without PFD					
Day of culture	Roots		Medium aqueous phase		Total		Roots		Medium aqueous phase		Total	
35	122.70 ± 16.49^a,d^		6.30 ± 2.29^b^		129.00 ± 17.21^c,e,w^		–		–		–	
42	257.94 ± 50.23^a,t^		14.28 ± 2.02^b,v^		272.22 ± 51.70^c,u,x^		248.46 ± 22.25		13.24 ± 1.79^y^		261.70 ± 23.95	
49	–		–		–		350.65 ± 72.56		13.21 ± 2.94		363.86 ± 75.28	

Culture variant	Single-elicited cultures with PFD-aerated						Twice-elicited cultures with PFD-aerated					
Day of culture	Roots	Medium aqueous phase		PFD phase		Total	Roots	Medium aqueous phase		PFD phase		Total
35	248.51 ± 69.12^d^	13.04 ± 3.82		5.62 ± 1.54		267.17 ± 73.69^e^	–	–		–		–
42	387.24 ± 124.97	27.30 ± 12.29		11.49 ± 7.50		426.02 ± 133.51	221.39 ± 63.34	4.66 ± 1.94		11.67 ± 7.02		237.71 ± 71.51
49	–	–		–		–	352.46 ± 120.14	10.51 ± 5.88		24.79 ± 5.20		387.77 ± 128.49

Culture variant	Single-elicited cultures with PFD-degassed						Twice-elicited cultures with PFD-degassed					
Day of culture	Roots	Medium aqueous phase		PFD phase		Total	Roots	Medium aqueous phase		PFD phase		Total
35	173.59 ± 49.21^f^	11.96 ± 4.04		6.46 ± 1.05^g,n,o^		192.00 ± 52.19^h,r^	–	–		–		–
42	477.02 ± 28.53^f,k,t^	12.98 ± 2.12^l,m^		13.11 ± 3.08^g,p^		503.11 ± 29.88^h,s,u^	274.43 ± 24.24^i,k^	4.98 ± 1.21^l,y^		9.64 ± 1.58^n^		289.05 ± 26.67^j,r,w^
49	–	–		–		–	381.70 ± 28.97^i^	8.85 ± 1.42^m,v^		28.41 ± 8.02^o,p^		418.96 ± 34.19^j,s,x^

Data represents mean of three replicates ± SD; the same letters indicate means statistically different (*p* < 0.05)

–, not applicable

**Table 3:** Paclitaxel content [µg/flask] determined in *Taxus* × *media* KT hairy root line cultures subjected to elicitation

Culture variant	Control cultures															
Day of culture	Roots						Medium aqueous phase						Total			
14	1.26 ± 0.02^a^						0.47 ± 0.34						1.74 ± 0.36^d^			
28	1.78 ± 0.20^a,b^						0.37 ± 0.07^b,f^						2.07 ± 0.11^l^			
35	2.17 ± 0.38°						1.39 ± 0.60						3.56 ± 0.72^d,m,p^			
42	2.75 ± 0.18^a,c,e,v^						0.22 ± 0.30^c,t^						2.87 ± 0.23^d,g,u,y,æ^			
49	1.28 ± 0.17						0.80 ± 0.35						2.03 ± 0.54^n,*^			

Culture variant	Unelicited cultures with PFD-aerated										Unelicited cultures with PFD-degassed					
Day of culture	Roots		Medium aqueous phase			PFD phase				Total	Roots	Medium aqueous phase		PFD phase		Total
28	4.94 ± 2.57		0.07 ± 0.01^f^			nd				6.09 ± 2.47	1.26 ± 0.08^h,Ş^	nd		nd		1.26 ± 0.08^j,Ş^
35	2.14 ± 1.96		nd			nd				2.14 ± 1.96	1.18 ± 0.20^hγ^	nd		nd		1.18 ± 0.20^j,lγ^
42	1.89 ± 0.32^e,β,α^		nd			nd				1.89 ± 0.32^g,β,α^	1.27 ± 0.51^h,Δ^	nd		nd		1.27 ± 0.51^j,m,Δ^
49	2.24 ± 0.94^i^		nd			nd				2.24 ± 0.94^k^	5.39 ± 1.61^h,i^	nd		nd		5.39 ± 1.61^j,k,n^

Culture variant	Single-elicited cultures without PFD										Twice-elicited cultures without PFD					
Day of culture	Roots			Medium aqueous phase				Total			Roots		Medium aqueous phase		Total	
35	17.13 ± 1.29^o,x,γ^			nd				17.13 ± 1.29^p,z,γ^			–		–		–	
42	nd			nd				nd			2.15 ± 0.20^r^		6.73 ± 0.06^r,t^		8.88 ± 0.24^s,u^	
49	–			–				–			3.31 ± 0.90		nd		3.31 ± 0.09^s^	

Culture variant	Single-elicited cultures with PFD-aerated										Twice-elicited cultures with PFD-aerated					
Day of culture	Roots	Medium aqueous phase			PFD phase				Total		Roots	Medium aqueous phase		PFD phase		Total
35	3.23 ± 0.20^w,x^	nd			nd				3.23 ± 0.20^y,z,λ^		–	–		–		–
42	50.51 ± 5.00^w,z,β^	nd			nd				50.51 ± 5.00^y,z,δ,β^		29.93 ± 4.30^z,v,α^	nd		nd		29.93 ± 4.30^z,æ,α^
49	–	–			–				–		nd	nd		nd		nd

Culture variant	Single-elicited cultures with PFD-degassed										Twice-elicited cultures with PFD-degassed					
Day of culture	Roots	Medium aqueous phase			PFD phase				Total		Roots	Medium aqueous phase		PFD phase		Total
35	6.93 ± 0.79^q,Ş^	nd			nd				6.93 ± 0.79^q,Ş,λ^		–	–		–		–
42	0.37 ± 0.11^q,Δ^	nd			nd				0.37 ± 0.11^q,δ,Δ^		29.86 ± 15.12	nd		0.53 ± 0.06		30.39 ± 15.11
49	–	–			–				–		22.47 ± 7.84	nd		nd		22.47 ± 7.84*

Data represents mean of three replicates ± SD; the same letters indicate means statistically different (*p* < 0.05)

–, not applicable, nd, not detected

**Table 4:** Paclitaxel and baccatin III content [µg/g DW] in two lines of *Taxus* × *media* hairy roots cultivated under various culture conditions

Culture variant	ATMA hairy root line (carrying *TXS* transgene)		KT hairy root line (without *TXS* transgene)	
Day of culture	Paclitaxel	Baccatin III	Paclitaxel	Baccatin III
Control				
14	nd	nd	18.33 ± 5.61^i^	nd
28	nd	nd	22.01 ± 7.92	nd
35	nd	nd	23.78 ± 1.84^n,o,u^	nd
42	nd	nd	28.80 ± 3.30^i,j,k,p,s,w^	nd
49	nd	nd	12.52 ± 1.89^i,j,x^	nd
Unelicited cultures with PFD-aerated				
28	nd	nd	50.15 ± 23.49	nd
35	nd	nd	20.75 ± 19.48^o,u^	nd
42	nd	nd	17.74 ± 3.03^k,s,w^	nd
49	nd	nd	21.45 ± 8.55^m,x^	nd
Unelicited cultures with PFD-degassed				
28	nd	nd	14.70 ± 5.50	nd
35	nd	nd	14.16 ± 2.13^n,o,u^	nd
42	nd	nd	18.69 ± 6.24^l,s^	nd
49	nd	nd	64.91 ± 25.34^l,m,x^	nd
Single-elicited cultures without PFD				
35	780.43 ± 136.74^a,e,^*	nd	164.62 ± 9.12^o,u,^*	nd
42	1570.14 ± 134.2^a,b,d,g^	nd	nd	nd
Twice-elicited cultures without PFD				
42	1259.86 ± 117.93^b,d,g,^*	nd	22.87 ± 1.58^p,s,w,^*	nd
49	1611.65 ± 228.96^h^	nd	nd	nd
Single-elicited cultures with PFD-aerated				
35	1292.21 ± 99.77^c,e,^*	nd	51.56 ± 15.29^r,^*	nd
42	2136.88 ± 266.83^c,d,g,^*	2.76 ± 0.62	470.08 ± 25.15^r,s,w,^*	nd
Twice-elicited cultures with PFD-aerated				
42	1246.07 ± 157.95^g,^*	1.91 ± 0.39	320.51 ± 33.24^s,^*	nd
49	1641.83 ± 509.17	6.58 ± 2.20	nd	nd
Single-elicited cultures with PFD-degassed				
35	1038.42 ± 288.28^f,^*	nd	67.12 ± 7.04^t,u,^*	nd
42	2473.29 ± 263.85^f,g,^*	2.32 ± 0.32	3.94 ± 1.17^s,t,w,^*	nd
Twice-elicited cultures with PFD-degassed				
42	1577.22 ± 347.18^g,^*	2.62 ± 0.72	401.02 ± 164.25*	nd
49	2056.19 ± 79.60^h,^*	4.70 ± 1.39	249.08 ± 62.84^x,^*	nd

Data presents mean ± SD. The same letters within columns indicate means statistically different (*p* < 0.05), while “*” indicates means statistically different (*p* < 0.05) within rows

The highest value of the total (intra- and extracellular) paclitaxel yield was determined in ATMA hairy root cultures in single-elicited PFD-degassed variant, after 2 weeks of elicitation, and amounted to 503.11 ± 29.88 µg/flask which corresponds to 2473.29 ± 263.85 µg/g DW (Tables 2, 4). This paclitaxel content was significantly higher than the value noted in elicited variants without PFD, with PFD-aerated and also twice-elicited PFD-degassed supported variants. The highest baccatin III amount [although not statistically significant (*p* > 0.05)] was noted in ATMA root line maintained in the presence of PFD-aerated and twice-elicited (6.58 ± 2.20 µg/g DW). The lowest paclitaxel content in ATMA roots was noticed in single-elicited cultures, but without the PFD, and it was almost twofold lower than the highest value (Tables 2, 4). In addition, only in culture variants without PFD phases, twice-repeated elicitation resulted in higher paclitaxel concentration than that observed in the single-elicited variant, although such difference was not statistically significant (*p* > 0.05). The extracellular level of paclitaxel detected in aqueous phase of culture medium and in PFD did not exceed 6.4% and 6.8% of its total concentration in single-elicited PFD-aerated and twice-elicited PFD-degassed variants (Table 2), respectively.

In KT roots, the highest total paclitaxel yield was achieved also in single-elicited cultures but only in variants supported with PFD-aerated 50.51 ± 5.00 µg/flask, what corresponds to 470.08 ± 25.15 µg/g DW (Tables 3, 4). In unelicited cultures supported with PFD-aerated or PFD-degassed the paclitaxel content was enhanced in comparison with the control, two and fivefold, respectively (Table 4). The single-elicitation strategy gave better results in culture variants without PFD and with PFD-aerated phases. Whereas twice-repeated elicitation, 1 week after first elicitor usage, was more effective in variants with PFD-degassed (Tables 3, 4). The extracellular presence of paclitaxel in KT root cultures was noted only in the control, unelicited variant with PFD-aerated and twice-elicited variants without PFD or with PFD-degassed (Table 3). In PFD phase, paclitaxel was detected solely in twice-elicited cultures supported with PFD-degassed (2% of its total content).

In the previous investigations on MJ, SNP, l-phenylanine and sucrose fed cultures of ATMA and ATM hairy root lines both carrying *TXS* transgene, solely paclitaxel was determined (Sykłowska-Baranek et al. 2015a) with its highest yield 1 week after elicitors treatment, while for the first time, paclitaxel was detected 24 h after treatment. Furthermore, in ATMA roots, the addition of MJ, l-phenylanine and sucrose without SNP to the two-phase cultures supported with PFD-aerated or degassed, paclitaxel and baccatin III were detected with their highest yield after 2 weeks of treatment—on the 42nd day (Sykłowska-Baranek et al. 2015b). The data are consistent with observations summarized by Nims et al. (2006) and Lenka et al. (2012) reporting first taxane accumulation at detectable levels 24 h after elicitation with their maximum production achieved from 2 to 15 days later (Nims et al. 2006). The data presented herein corroborate also with results obtained by Exposito et al. (2010) who found that after MJ addition to the production medium, the total taxane yield in TXS cell line was 1.6-fold higher than in RolC line (23.3 mg/l vs 15 mg/l). Moreover, the authors did not notice the changes in extracellular level of taxanes as a consequence of MJ addition to RolC cell line cultures, while in TXS cell line cultures, their secretion rose up to 54% from 15% on average. Under the conditions of the current study, the extracellular paclitaxel amount observed in ATMA root elicited cultures not exceeding 10% of its total content both in aqueous and PFD phases. While in KT root cultures, paclitaxel was released to the liquid phases of the medium mostly in control and unelicited cultures at 26% on average, although increased almost up to 76% in twice-elicited culture variant without PFD. In the presented study, the intracellular paclitaxel concentration increases 1.2-times upon the most favourable for taxane biosynthesis conditions (variant 7) in comparison with MJ, l-phenylalanine and sucrose fed cultures (Sykłowska-Baranek et al. 2015a) and 1.7-fold, where ATMA roots were cultivated under the above mentioned additives supplementation but in PFD-degassed supported cultures (Sykłowska-Baranek et al. 2015b).

Sabater-Jara et al. (2014) in TXS cell line cultures reported the synergistic effect of joint action of MJ and cyclodextrins, where cyclodextrins acting as an elicitor but also taking part in taxane complexation due to their physicochemical nature. Cyclodextrins are able to form inclusion complexes with the lipophilic centre that provides a microenvironment into which non-polar compounds could enter (Del Valle 2004) and are able to trap apolar taxanes. The entrapping of taxanes inside cyclodextrin inclusions could counteract the biosynthetic feedback inhibition, their degradation in the medium and the paclitaxel detrimental effect on the cell viability (Kim et al. 2005; Expósito et al. 2009). Perfluorochemicals, among them PFD, apart from their unique properties for dissolving large volumes of gases in ‘molecular cavities’ without a chemical reaction being involved (Lowe et al. 1998; Lowe 2002) and biological inertness are reported to be used as a drug delivery systems (Lehmler 2007) or in situ extractant for hydrophobic alkannin/shikonin compounds (Sykłowska-Baranek et al. 2014). PFD, under conditions of the current study, created an in situ extracting phase which could counteract the growth suppressing effect of accumulating in medium phase paclitaxel (Table 2). PFD did not act as elicitor, only as a product depository and did not itself influence taxane production (Tables 2, 3, 4).

Up to now, the repeated elicitation with fungal elicitor together with medium removal resulted in a 40% increase in paclitaxel yield in comparison with single-elicited culture in *T. chinensis* cell suspension cultures (Wang et al. 2001). In research by Qian et al. (2005), also in cell suspension cultures of *T. chinensis*, repeated elicitation with jasmonate analogue (2,3-dihydroxypropyl jasmonate) combined with additional sucrose feeding allowed to enhance 5.4-times the taxuyunnanine C production in relation with control untreated cultures. Despite these beneficial effects of repeated elicitation, jointly with either medium exchange or mid-cycle sucrose feeding, a similar strategy applied in the current investigations did not result in the enhancement of paclitaxel or other taxanes accumulation in comparison with single-elicited cultures.

### Gene expression profiles

To elucidate the molecular mechanism underlaying the diverse response of cultivated two *T*. × *media* hairy root lines subjected to elicitation with SNP and MJ, supplementation with l-phenylalanine and sucrose feeding in PFD-degassed supported cultures, the expression profile of *TXS, BAPT*, and *DBTNBT* genes was investigated after 6 h, 12 h, 24 h, 48 h, 1, and 2 weeks after elicitors addition. The *TXS* gene codifies taxadiene synthase involved in the first committed step of taxane biosynthesis, while *BAPT* and *DBTNBT* genes are engaged in the late pathway steps and are believed to be rate-limiting ones. In the PFD-degassed single-elicited cultures the highest paclitaxel content was determined in the hairy roots of ATMA line, while the lowest in the hairy roots of KT line. The transcript levels observed in elicited cultures were compared with transcript levels in control roots from the 28th day of culture which represents the timepoint of elicitor application. The proposed approach could allow to shed light on the molecular regulation underlaying considerably diverse levels of paclitaxel and baccatin III production in two *T*. × *media* hairy roots cultivated under conditions of the current study. Moreover, the investigation of gene expression profiles in twice-elicited PFD-degassed supported culture variant was to be performed; however, the quality of the isolated RNA was unsatisfactory (less than 1.9–2.1 260:280 ratio) to perform qRT-PCR analysis. This could be attributed to the enhanced phenolic compounds accumulation in twice-elicited roots. In addition, in ATMA hairy root cultures, no taxane accumulation was observed before elicitor treatment.

**Fig. 2:**
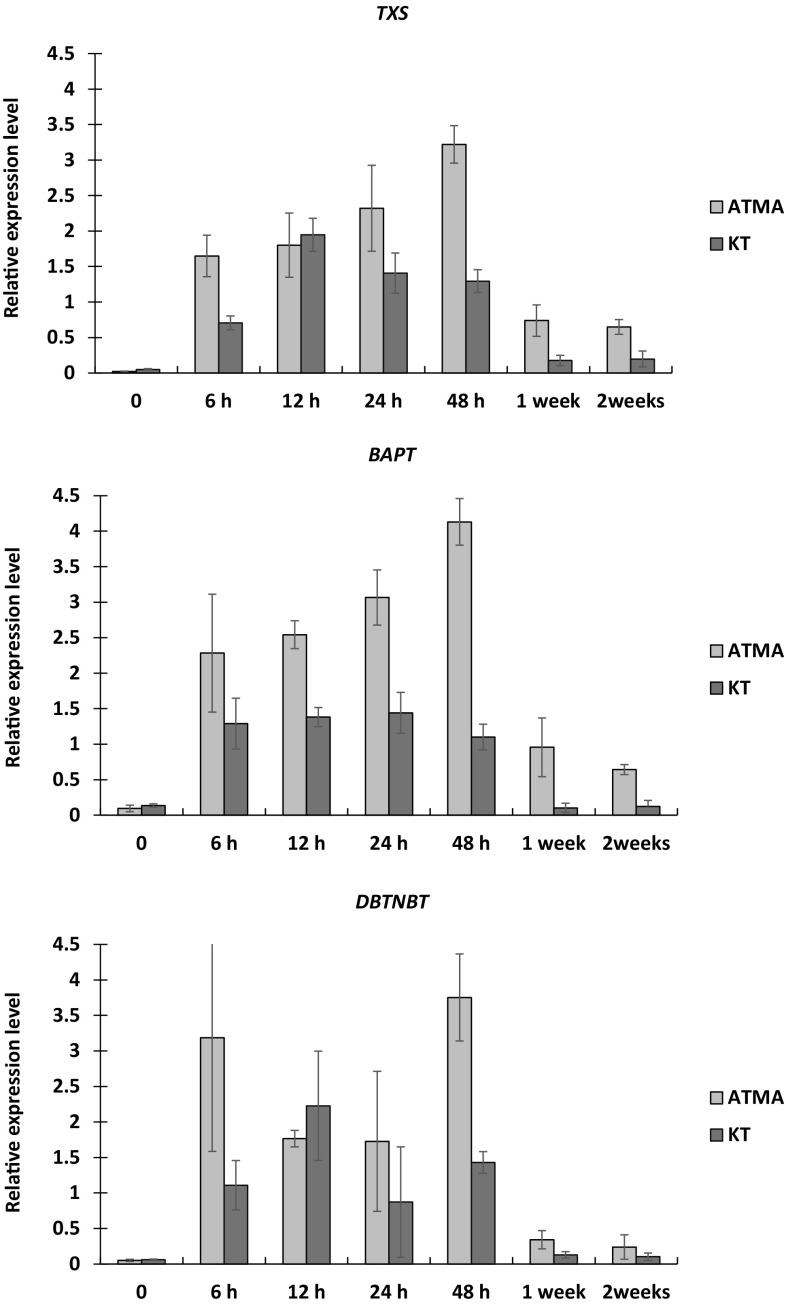
Relative expression levels of *TXS, BAPT*, and *DBTNBT* genes in *Taxus* × *media* two hairy root lines: ATMA (harbouring *TXS* transgene) and KT (without *TXS* transgene). Both hairy root lines were cultivated in two-phase PFD-degassed supported cultures elicited with MJ, SNP and supplemented with l-phenylalanine and additional sucrose. The relative gene expression levels in elicitor treated cultures was compared with their expression in untreated cultures on the 28th day (day of elicitors application) – time point “0”. The transcript level of each gene was normalized against reference genes: 18S rDNA and β-tubulin using ΔΔCt method with PCR efficiency correction. The reported values are an average of four biological replicates and three technical replicates ± SD

In both hairy root lines, all examined genes were expressed in control untreated cultures and were considerably induced by elicitor treatment (Fig. 2). At the time point of elicitation (the 28th day), the lowest expression level exhibited *TXS* gene in both root lines, although in KT roots, it was 2.4-fold higher than in ATMA roots. In ATMA roots, the highest *TXS* gene expression was noted 48 h after elicitation and followed by its decline (Fig. 1a), although at the end of the culture (the 42nd day, 2 weeks after elicitation), the *TXS* gene expression was still about 30-fold higher than before elicitor treatment. In KT roots, the highest expression of *TXS* gene was detected 12 h after elicitation and it was 1.7-fold lower than in ATMA at its highest abundance. Final *TXS* gene expression level was almost fourfold higher than the initial one.

Furthermore, the expression profiles of two genes *BAPT* and *DBTNBT* involved in the last steps of taxane biosynthesis were investigated. The *BAPT* gene expression in KT control roots on the 28th day was 1.4-fold higher that in ATMA control roots (Fig. 2b). *BAPT* highest expression in ATMA roots was detected 48-h post-elicitation, and was almost 43-fold higher than in the control. While in KT roots, the *BAPT* gene was the most expressed 24-h post-elicitation, although only 10.7-fold more than in the control. In control cultures of KT and ATMA root lines, the expression of the *DBTNBT* gene was nearly twofold lower than the *BAPT* gene. The expression levels of *TXS* and *DBTNBT* genes in KT roots were similar under control conditions, while in ATMA roots, the *DBTNBT* gene expression level was twofold higher in comparison with the expression level of the *TXS* gene. The highest, and 2.6-fold higher than in KT roots, the expression level of the *DBTNBT* gene was noticed in ATMA roots 48-h post-elicitation and it was about 75-fold more than in the control. In KT roots, the highest level of *DBTNBT* expression was observed 12-h post-elicitor treatment and represents a 38-fold increase in comparison with control (Fig. 2c). The expression of all genes investigated in the current study was lower in KT roots than in ATMA roots (significant statistically *p* > 0.05) which corresponds with the higher paclitaxel content in ATMA hairy root cultures.

In the current study, similar to the previous observations by Nims et al. (2006), the distinct shift in the highest expression of investigated genes and the occurrence of paclitaxel and baccatin III production was noted. Nims et al. (2006) noticed the expression of *TXS* gene in unelicited and MJ-elicited cell suspension cultures of *T. cuspidata* with the obvious increase in transcript levels upon MJ addition and its highest expression noted 18-h post-elicitation. The *BAPT* gene expression was low both in unelicited and elicited roots and its highest transcript level was noticed 6-h post-elicitation, while *DBTNBT* expression was undetectable without elicitor addition. Its transcript abundance increased 6 h after MJ application and remained at the parallel levels also at 18 h. Moreover, the authors reported the occurrence of non-side chain taxanes no earlier than 7-day post-elicitation although transcripts of *BAPT* and *DBTNBT* did not persist in 7-day cultures. It may indicate that *BAPT* and *DBTNBT* genes that catalyses the relevant enzymes of late biosynthetic pathway could limit the paclitaxel and other taxanes yield. Exposito et al. (2010) reported the differential expression pattern for *TXS* gene in RolC and TXS cell lines. MJ caused a rapid increase in its expression which peaked at 12-h post-elicitation in TXS line, while in RolC line its highest abundance was noted on the 2nd day. The expression level of *TXS* gene detected in the RolC cell line was twice of that in the TXS cell line which is opposite to the results of the current study, where the 1.7-fold lower *TXS* gene expression in KT than in ATMA hairy roots was observed. Moreover, the expression pattern of the *TXS* gene demonstrated in the current study was totally opposite to those reported by Exposito et al. (2010). In our study, the highest *TXS* gene expression was noted 2 days after elicitation in the KT roots, while in ATMA roots—12-h post-elicitation. This could indicate that the gene expression in various *Taxus* species is regulated also on the level of activation of different transcription factors which has been demonstrated recently by Lenka et al. (2015) and Sun et al. (2013) and the possible role of miRNAs was postulated (Sun et al. 2013). The discrepancies detected between gene expression patterns in cell suspension and hairy root cultures could be also attributed to the different growth and differentiation regulatory programmes in undifferentiated cell suspension cultures and differentiated, organizing organs (Flores et al. 1999). Although the results of the current experiment corresponds to those reported by Exposito et al. (2010) in respect to the capabilities for taxane production. In addition, under conditions of the current study, the hairy roots harbouring *TXS* transgene (ATMA line) were characterized by higher production potential than those carrying solely *ROL* genes (KT line). Onrubia et al. (2013b) in *T*. × *media* cell suspension cultures carrying *TXS* transgene, exhibited the notable increase in *TXS* gene expression 24-h post-MJ elicitation which was sustained furtherly on days 2 and 4. While under coronatine treatment *TXS* gene transcript levels peaked on day 2 and declined thereafter. Overall the *TXS* gene transcript abundance was higher under MJ than coronatine treatment, although taxane content was considerably higher under the influence of coronatine.

As far as the expression of *BAPT* and *DBTNBT* genes were considered in *T*. × *media* cell line harbouring *TXS* transgene, their expression levels were higher under MJ elicitation, whereas the total taxane content and specifically paclitaxel was higher under coronatine elicitation (Onrubia et al. 2013b). Furthermore, Sabater-Jara et al. (2014) in cell suspension cultures of *T*. × *media* harbouring *TXS* transgene, comparing the influence of simultaneous elicitation of MJ and randomly methylated-β-cyclodextrins (M-β-CD) to elicitation performed with MJ or M-β-CD separately, noted a significant increase in, among others, *TXS, BAPT* and *DBTNBT* expression levels and taxane production than in the control and separately elicited cultures. Although also the shift in their highest expression and the highest paclitaxel production was observed which is in accordance with previous reports by Nims et al. (2006), Exposito et al. (2010), Onrubia et al. (2013b) and results of the current study. In *T. baccata* cell suspension cultures the molecular mechanism underlaying the activity of the joint action of MJ and vanadyl sulphate (VS) revealed that MJ induced unambiguously the activity of *TXS* and *BAPT* genes and taxane production. The influence of the combined application of MJ and VS was not so effective on production enhancement which was also demonstrated on the basis of gene expression (Onrubia et al. 2010). Although joint action of MJ and VS and MJ and M-β-CD was investigated on the levels of the elicitor treatment influencing gene expression nothing is known on the effect of SNP and MJ combined activity and what’s more in a two-phase supported PFD culture systems. In current investigations it was observed the co-increased expression of the three examined genes *TXS, BAPT* and *DBTNBT* in hairy roots which is opposite to their expression pattern determined in *T. baccata* seedling roots (Onrubia et al. 2011). Although the authors also noted an expression co-increase of these genes but in the aerial parts of the seedlings, and their results indicates that the main role in biosynthesis of paclitaxel seemed to be played by the *DBTNBT* gene. The examination of *TXS* gene expression levels in hairy roots subjected to the current investigation, confirms the observation by Hezari et al. (1997) that this gene is not rate-limiting in the taxane biosynthesis pathway. However, the activity of *TXS* synthase supplies the sufficient quantities of the intermediated product for the further step in taxane biosynthesis which was also observed under the conditions of the current study. Nevertheless, the *TXS* gene harbouring cell lines (Exposito et al. 2010) and hairy roots under conditions of the current study produced significantly (*p* < 0.05) higher quantities of taxanes than cell or hairy root lines without this transgene. The rate-limiting step in taxane biosynthesis seems to be the activity of the *DBAT* gene which codes for the enzyme responsible for the conversion of 10-deacetylbaccatin III to baccatin III (Onrubia et al. 2011; Patil et al. 2012; Nasiri et al. 2016). Under conditions of current study 10-deacetylbaccatin III was at undetectable levels in both investigated hairy root lines and only very low amounts of baccatin III were detected solely in ATMA root lines after elicitor treatment. Our results seems to support the suggestion by Lenka et al. (2012) that the taxane biosynthesis capacity is controlled rather by genes induced early post-MJ addition, but genes not only involved in the biosynthesis itself but engaged in the total metabolism. Moreover, in *T. chinensis* two jasmonate-responsive elements acting in the opposite way were discovered (Zhang et al. 2015). Among them the TcERF12 acted as the repressor, while TcRF15 as an activator of *TXS* gene expression. Furthermore, Lenka et al. (2015) reported that TcJAMYC1, TcJAMYC2 TcJAMYC4 transcription factors of *T. cuspidata* negatively regulates the promoters of *BAPT* and *DBTNBT* genes, whereas not affecting the activity of *TXS* gene. Nims et al. (2006) first indicted that although the expression level of *BAPT* and *DBTNBT* genes did not exist 7-day post-MJ elicitation, the relevant enzyme activity may persist long after the quenching of genes expression. This suggestion was furtherly confirmed by Exposito et al. (2010) who noted the activity of taxadiene synthase until the end of culture (day 28). The results of current study are also supported by data obtained from transcript profiling experiments performed in cell suspension cultures of various *Taxus* spp. which points to and confirms the complex nature of yew metabolism regulation (Lenka et al. 2012, 2015; Li et al. 2012; Sun et al. 2013).

## Conclusions

For two independent lines of *T*. × *media* hairy roots: ATMA (harbouring *TXS* transgene) and KT (without *TXS* transgene), the effect of repeated elicitation with mixture of MJ and SNP, mid-cycle PHEN and sucrose feeding in single and two-phase culture variants was investigated. Moreover, the molecular mechanism underlaying the response of two genetically different hairy root lines to culture conditions was examined. The most influencing and statistically significant factor determinative for the taxane production output seems to be the duration of elicitation, because the maximal value of taxane accumulation was obtained after 2 weeks of elicitation. The proposed repeated elicitation strategy proved not to be the most effective approach for enhance taxane accumulation. The two-phase culture systems composed with aqueous culture medium and PFD were identified as the crucial factor for improvement of paclitaxel production. The highest paclitaxel content was determined in ATMA roots cultured in single-elicited variant in two-phase culture system with PFD-degassed. In both investigated hairy root lines the gene expression pattern differed substantially, and such effect reflected the taxane output. The highest observed co-expression of *TXS, BAPT* and *DBTNBT* genes 48-h post-elicitation in ATMA roots corresponded with higher taxane production in cultures of this root line in comparison with KT roots. In KT roots the gene expression level was notably lower than in ATMA roots. The up-regulation of three investigated genes coherently indicates that *TXS* together with previously suggested to be rate-limiting in taxane biosynthesis *BAPT* and *DBTNBT* genes, are not the unique factors regulating the complex nature of *Taxus* spp. responses to environmental stimuli. The results of the current study, also support and strengthen the hypothesis that the molecular and metabolic effects of culture conditions greatly depends on the genetic properties of in vitro cultured *Taxus* cells, tissues or organs, as well as on culture medium/system composition, type and mode of elicitor treatment.

### Author contribution statement

KSB planning of the investigations, carrying out cultures of KT hairy root lines, RNA isolation, HPLC-UV-DAD analysis, elaboration of the results and preparation of the manuscript. WR elaboration of method of RNA isolation and gene expression, elaboration of results from gene expression analysis. MG cleaning of samples resulting from KT hairy root cultures, performing gene expression analysis. PR carrying out cultures of ATMA hairy root lines, sample preparation, elaboration of results. MP experiments with in situ applied PFD, results elaboration and discussion, critical reading of the manuscript. MGB design of primer sequences and preliminary investigations on gene expression. JH results elaboration and discussion, critical reading of the manuscript. AP results elaboration and discussion, critical reading of the manuscript. All authors read and approved the final version of the manuscript.

## Electronic supplementary material

Below is the link to the electronic supplementary material.

Supplementary material 1 (DOCX 14 KB)

## Abbreviations

ATMA: Hairy root line harbouring taxadiene synthase transgene
KT: Hairy root without *TXS* transgene
*TXS*: Taxadiene synthase
*BAPT*: Baccatin III: 3-amino, 3-phenylpropanoyltransferase
*DBTNBT*: 3′-*N*-debenzoyl-2-deoxytaxol-*N*-benzoyltransferase
PFD: Perfluorodecalin
PFCs: Liquid perfluorochemicals (syn. perfluorocarbons)
FW: Fresh weight
DW: Dry weight
MJ: Methyl jasmonate
SNP: Sodium nitroprusside
VS: Vanadyl sulphate
M-β-CD: Randomly methylated-β-cyclodextrin

## References

[CR1] Cai Z, Kastell A, Knorr D, Smetanska I (2012). Exudation: an expanding technique for continuous production and release of secondary metabolites from plant cell suspension and hairy root cultures. Plant Cell Rep.

[CR2] Chomczynski P, Sacchi N (1987). Single-step method of RNA isolation by acid guanidinium thiocyanate-phenol-chloroform extraction. Anal Biochem.

[CR49] Castro CI, Briceno JC (2010). Perfluorocarbon-based oxygen carriers: review of products and trials. Artif Organs.

[CR3] Cusido RM, Onrubia M, Sabater-Jara AB, Moyano E, Bonfill M, Goossens A, Pedreño MA, Palazon J (2014). A rational approach to improving the biotechnological production of taxanes in plant cell cultures of *Taxus* spp. Biotechnol Adv.

[CR4] Del Valle EMM (2004). Cyclodextrins and their uses: a review. Process Biochem.

[CR5] Exposito O, Syklowska-Baranek K, Moyano E, Onrubia M, Bonfill M, Palazon J, Cusido RM (2010). Metabolic responses of *Taxus media* transformed cell cultures to the addition of methyl jasmonate. Biotechnol Prog.

[CR6] Expósito O, Bonfill M, Onrubia M, Jané A, Moyano E, Cusidó RM, Palazón J, Piñiol T (2009). Effect of taxol feeding on taxol and related taxane production in *Taxus baccata* suspension cultures. New Biotechnol.

[CR7] Flores H, Vivanco J, Loyola-Vargas V (1999). “Radicle” biochemistry: the biology of root-specific metabolism. Trends Plant Sci.

[CR50] Frense D (2007). Taxanes: perspectives for biotechnological production. Appl Microbiol Biotechnol.

[CR8] Furmanowa M, Sykłowska-Baranek K (2000). Hairy root cultures of T*axus*×*media var. Hicksii* Rehd. as a new source of paclitaxel and 10-deacetylbaccatin III. Biotechnol Lett.

[CR9] Hezari M, Ketchum RE, Gibson DM, Croteau R (1997). Taxol production and taxadiene synthase activity in *Taxus canadensis* cell suspension cultures. Arch Biochem Biophys.

[CR10] Kim BJ, Gibson DM, Shuler ML (2004). Effect of subculture and elicitation on instability of taxol production in *Taxus* sp. suspension cultures. Biotechnol Prog.

[CR11] Kim BJ, Gibson DM, Shuler ML (2005). Relationship of viability and apoptosis to taxol production in *Taxus* sp. suspension cultures elicited with methyl jasmonate. Biotechnol Prog.

[CR12] Lehmler H-J (2007). Perfluorocarbon compounds as vehicles for pulmonary drug delivery. Expert Opin Drug Deliv.

[CR13] Lenka SK, Boutaoui N, Paulose B, Vongpaseuth K, Normalny J, Roberts SC, Walker EL (2012). Identification and expression analysis of methyl jasmonate responsive ESTs in paclitaxel producing *Taxus cuspidata* suspension culture cells. BMC Genom.

[CR14] Lenka SK, Nims NE, Vongpaseuth K, Boshar RA, Roberts SC, Walker EL (2015). Jasmonate-responsive expression of paclitaxel biosynthesis genes in *Taxus cuspidata* cultured cells is negatively regulated by the bHLH transcription factors TcJAMYC1, TcJAMYC2, and TcJAMYC4. Front Plant Sci.

[CR15] Li S, Zhang P, Zhang M, Fu C, Zhao C, Dong Y, Guo A, Yu L (2012). Transcriptional profile of *Taxus chinensis* cells in response to methyl jasmonate. BMC Genom.

[CR16] Lowe KC (2002). Perfluorochemical respiratory gas carriers: benefits to cell culture systems. J Fluor Chem.

[CR17] Lowe KC, Davey MR, Power JB (1998). Perfluorochemicals: their applications and benefits to cell culture. Trends Biotechnol.

[CR18] Maheshwari P, Garg S, Kumar A (2008). Taxoids: biosynthesis and in vitro production. Mol Biol.

[CR19] Malik S, Cusidó RM, Mirjalili MH, Moyano E, Palazón J, Bonfill M (2011). Production of the anticancer drug taxol in *Taxus baccata* suspension cultures: a review. Process Biochem.

[CR20] Malik S, Mirjalili MH, Fett-Neto AG, Mazzafera P, Bonfill M (2013). Living between two worlds: two-phase culture systems for producing plant secondary metabolites. Crit Rev Biotechnol.

[CR21] Nasiri J, Naghavi MR, Alizadeh H, Moghadam MRF (2016). Seasonal-based temporal changes fluctuate expression patterns of *TXS, DBAT, BAPT* and *DBTNBT* genes alongside production of associated taxanes in *Taxus baccata*. Plant Cell Rep.

[CR22] Nims E, Dubois CP, Roberts SC, Walker EL (2006). Expression profiling of genes involved in paclitaxel biosynthesis for targeted metabolic engineering. Metab Eng.

[CR23] Onrubia M, Moyano E, Bonfill M, Expósito O, Palazón J, Cusidó RM (2010). An approach to the molecular mechanism of methyl jasmonate and vanadyl sulphate elicitation in *Taxus baccata* cell cultures: the role of *txs* and *bapt* gene expression. Biochem Eng J.

[CR24] Onrubia M, Moyano E, Bonfill M, Palazón J, Goossens A, Cusidó RM (2011). The relationship between *TXS. DBAT, BAPT* and *DBTNBT* gene expression and taxane production during the development of *Taxus baccata* plantlets. Plant Sci.

[CR25] Onrubia M, Cusidó RM, Ramirez K, Hermández-Vázquez L, Moyano E, Bonfill M, Palazon J (2013). Bioprocessing of plant in vitro systems for the mass production of pharmaceutically important metabolites: paclitaxel and its derivatives. Curr Med Chem.

[CR26] Onrubia M, Moyano E, Bonfill M, Cusidó RM, Goossens A, Palazón J (2013). Coronatine, a more powerful elicitor for inducing taxane biosynthesis in *Taxus media* cell cultures than methyl jasmonate. J Plant Physiol.

[CR27] Patil RA, Kolewe ME, Normanly J, Walker EL, Roberts SC (2012). Contribution of taxane biosynthetic pathway gene expression to observed variability in paclitaxel accumulation in *Taxus* suspension cultures. Biotechnol J.

[CR28] Patil RA, Lenka SK, Normanly J, Walker EL, Roberts SC (2014). Methyl jasmonate represses growth and affects cell cycle progression in cultured *Taxus* cells. Plant Cell Rep.

[CR29] Pilarek M (2014). Liquid perfluorochemicals as flexible and efficient gas carriers applied in bioprocess engineering: an updated overview and future prospects. Chem Process Eng.

[CR30] Qian Z-G, Zhao Z-J, Xu Y, Qian X, Zhong J-J (2005). Highly efficient strategy for enhancing taxoid production by repeated elicitation with a newly synthesized jasmonate in fed-batch cultivation of *Taxus chinensis* cells. Biotechnol Bioeng.

[CR31] Sabater-Jara AB, Tudela LR, López-Pérez AJ (2010). In vitro culture of *Taxus* sp.: strategies to increase cell growth and taxoid production. Phytochem Rev.

[CR32] Sabater-Jara AB, Onrubia M, Moyano E, Bonfill M, Palazón J, Pedreño MA, Cusidó RM (2014). Synergistic effect of cyclodextrins and methyl jasmonate on taxane production in *Taxus*×*media* cell cultures. Plant Biotechnol J.

[CR33] Sobieszuk P, Pilarek M (2012). Absorption of CO_2_ into perfluorinated gas carrier in the Taylor gas–liquid flow in a microchannel system. Chem Process Eng.

[CR34] Sun G, Yang Y, Xie F, Wen J, Wu J, Wilson IW, Tang Q, Liu H, Qiu D (2013). Deep sequencing reveals transcriptome re-programming of *Taxus* × *media* cells to the elicitation with methyl jasmonate. PLoS One.

[CR35] Syklowska-Baranek K, Pietrosiuk A, Kokoszka A, Furmanowa M (2009). Enhancement of taxane production in hairy root culture of *Taxus*×*media var. Hicksii*. J Plant Physiol.

[CR36] Sykłowska-Baranek K, Pilarek M, Cichosz M, Pietrosiuk A (2014). Liquid perfluorodecalin application for in situ extraction and enhanced naphthoquinones production in *Arnebia euchroma* cell suspension cultures. Appl Biochem Biotechnol.

[CR37] Sykłowska-Baranek K, Grech-Baran M, Naliwajski MR, Bonfill M, Pietrosiuk A (2015). Paclitaxel production and PAL activity in hairy root cultures of *Taxus*×*media var. Hicksii* carrying a taxadiene synthase transgene elicited with nitric oxide and methyl jasmonate. Acta Physiol Plant.

[CR38] Sykłowska-Baranek K, Pilarek M, Bonfill M, Kafel K, Pietrosiuk A (2015). Perfluorodecalin-supported system enhances taxane production in hairy root cultures of T*axus*×*media var. Hicksii* carrying a taxadiene synthase transgene. Plant Cell Tissue Organ Cult.

[CR39] Tabata H (2004). Paclitaxel production by plant-cell-culture technology. Adv Biochem Eng Biotechnol.

[CR40] Theodoridis G, de Jong CF, Laskaris G, Verpoorte R (1998). Application of SPE for the HPLC analysis of taxanes from *Taxus* cell cultures. Chromatographia.

[CR41] Theodoridis G, Laskaris G, de Jong CF, Hofte AJP, Verpoorte R (1998). Determination of paclitaxel and related diterpenoids in plant extracts by high-performance liquid chromatography with UV detection in high-performance liquid chromatography-mass spectrometry. J Chromatogr A.

[CR42] Vongpaseuth K, Roberts SC (2007). Advancements in the understanding of paclitaxel metabolism in tissue culture. Curr Pharm Biotechnol.

[CR43] Walker K, Fujisaki S, Long R, Croteau R (2002). Molecular cloning and heterologous expression of the C-13 phenylpropanoid side chain-CoA acyltransferase that functions in taxol biosynthesis. Proc Natl Acad Sci USA.

[CR44] Walker K, Long R, Croteau R (2002). The final acylation step in taxol biosynthesis: cloning of the taxoid C13-side-chain *N*-benzoyltransferase from Taxus. Proc Natl Acad Sci.

[CR45] Wang C, Wu J, Mei X (2001). Enhanced taxol production and release in *Taxus chinensis* cell suspension cultures with selected organic solvents and sucrose feeding. Biotechnol Prog.

[CR46] Wilson SA, Roberts SC (2012). Recent advances towards development and commercialization of plant cell culture processes for the synthesis of biomolecules. Plant Biotechnol J.

[CR47] Yared JA, Tkaczuk KHR (2012). Update on taxane development: new analogs and new formulations. Drug Des Devel Ther.

[CR48] Zhang M, Li S, Nie L, Chen Q, Xu X, Yu L, Fu C (2015). Two jasmonate-responsive factors, TcERF12 and TcERF15, respectively act as repressor and activator of tasy gene of taxol biosynthesis in *Taxus chinensis*. Plant Mol Biol.

