# Supplementary material for: Comparison of elicitor-based effects on metabolic responses of Taxus × media hairy roots in perfluorodecalin-supported two-phase culture system
Source: Plant Cell Rep. 2018 Nov 7;38(1):85–99. doi: 10.1007/s00299-018-2351-0 (PMC6320355; doi:10.1007/s00299-018-2351-0)
Supplement: Supplementary file 1 — Supplementary material 1 (DOCX 14 KB) [file 299_2018_2351_MOESM1_ESM.docx]

**Table 1S Experiment design**

| **Day of culture** | **Control** | **Variant 1** | **Variant 2** | **Variant 3** | **Variant 4** | **Variant 5** | **Variant 6** | **Variant 7** | **Variant 8** |
| --- | --- | --- | --- | --- | --- | --- | --- | --- | --- |
|  | **Untreated cultures** | **Single elicited cultures without PFD** | **Twice elicited cultures without PFD** | **Unelicited cultures with PFD-aerated** | **Single elicited cultures with PFD-aerated** | **Twice elicited cultures with PFD-aerated** | **Unelicited cultures with PFD-degassed** | **Single elicited cultures with PFD-degassed** | **Twice elicited cultures with PFD-degassed** |
| **Day 0** | inoculation | inoculation | inoculation | inoculation | inoculation | inoculation | inoculation | inoculation | inoculation |
| **Day 14** | sample harvesting | - | - | PFD application | PFD application | PFD application | PFD application | PFD application | PFD application |
| **Day 28** | sample harvesting | elicitation | elicitation | sample harvesting | elicitation | elicitation | sample harvesting | elicitation | elicitation |
| **Day 35** | sample harvesting | sample harvesting | elicitation | sample harvesting | sample harvesting | elicitation | sample harvesting | sample harvesting | elicitation |
| **Day 42** | sample harvesting | sample harvesting - end of the culture | sample harvesting | sample harvesting | sample harvesting - end of the culture | sample harvesting | sample harvesting | sample harvesting - end of the culture | sample harvesting |
| **Day 49** | sample harvesting – end of the culture | - | sample harvesting – end of the culture | sample harvesting – end of the culture | - | sample harvesting – end of the culture | sample harvesting – end of the culture | - | sample harvesting – end of the culture |
